# *Camelina sativa* Seeds and Oil as Ingredients in Model Muffins in Order to Enhance Their Health-Promoting Value

**DOI:** 10.3390/foods13132027

**Published:** 2024-06-26

**Authors:** Agnieszka Bilska, Danuta Kurasiak-Popowska, Tomasz Szablewski, Monika Radzimirska-Graczyk, Kinga Stuper-Szablewska

**Affiliations:** 1 Department of Food and Nutrition, Poznan University of Physical Education, Królowej Jadwigi 27/39, 61-871 Poznan, Poland; graczyk@awf.poznan.pl; 2 Department of Genetics and Plant Breeding, Faculty of Agriculture, Horticulture and Biotechnology, Poznan University of Life Sciences, ul. Dojazd 11, 60-632 Poznan, Poland; danuta.kurasiak-popowska@up.poznan.pl; 3 Department of Food Quality and Safety Management, Faculty of Food Science and Nutrition, Poznan University of Life Sciences, Wojska Polskiego 31, 60-624 Poznan, Poland; tomasz.szablewski@up.poznan.pl; 4 Department of Chemistry, Faculty of Forestry and Wood Technology, Poznan University of Life Sciences, ul. Wojska Polskiego 75, 60-625 Poznan, Poland; kinga.stuper@up.poznan.pl

**Keywords:** bioactive compounds, food products, muffins, *Camelina sativa*, seeds, oil, phenolic acids, carotenoids

## Abstract

The aim of this study was to see whether it is possible to add camelina oil and seeds as ingredients in muffins in order to enhance their health-promoting value, such as their bioactive compound content, while maintaining the organoleptic attributes considered desirable by consumers. Camelina oil is characterised by a high linolenic acid content. Four types of muffins were prepared for analysis: M*Bn*O—control muffins (containing 11.85% rapeseed oil), M*Cs*O—muffins containing camelina oil instead of rapeseed oil, M*Cs*S—muffins containing 6.65% camelina seeds in relation to the mass of prepared dough, and M*Cs*OS—muffins containing both camelina oil and camelina seeds. The change in the fatty acid profile in muffins with the addition of camelina oil was significant; however, it was found that, as a result of thermal treatment, lower amounts of saturated fatty acids were formed. Among all the investigated experimental variants, muffins were characterised by the highest contents of all the phenolic acids analysed. The substitution of rapeseed oil with camelina oil had no negative effect on most of the organoleptic attributes of the muffins. Moreover, thanks to a greater content of carotenoids, camelina oil had an advantageous effect on the improvement of product colour, thus improving its overall desirability.

## 1. Introduction

As a result of civilisational progress and accelerating industrialisation, we have been observing a rapid increase in the number of factors posing a direct health hazard. The most numerous of these factors is the so-called lifestyle diseases, which lead to enormous burden and substantial costs for the healthcare system [1]. A key risk factor in the development of lifestyle diseases is related to oxidative stress, resulting from an excessive generation of reactive oxygen species (ROS), which are not eliminated by natural repair mechanisms. Numerous studies indicate that cell repair mechanisms may be promoted by the intake of substances exhibiting antioxidant activity [2,3,4]. Plant origin materials are particularly rich sources of these substances [5,6,7,8,9,10]. Current recommendations and trends in rational nutrition are aimed at increasing the share of non-processed plant origin foodstuffs in the daily diet [11,12,13]. In view of the sensory attributes of some raw materials of plant origin, which are considered unacceptable by consumers, the development of novel products with high health-promoting value containing plant materials rich in bioactive compounds has been attempted.

Thermal processing of food is a key technological process providing conditions for the formation of a wide array of new chemicals affecting the sensory attributes of food. Daily diet is a primary source of the Maillard reaction products formed in food stuffs as a consequence of technological factors. An equally important role is played by raw material parameters, such as the presence of antioxidants as potential inhibitors of that reaction. It has been observed that phenolic compounds exhibiting strong free radical scavenging activity may be used to prevent the formation of undesirable Maillard reaction products. Saturated fatty acids also account for a high share of the contemporary human diet. For this reason, from a dietary point of view, it is crucial to enrich the diet with foodstuffs containing mono- and polyunsaturated fatty acids supplied at appropriate ratios [14].

It is very difficult to replace certain ingredients in confectionary products, since this may result in changes of organoleptic properties in the final product, which is undesirable from the consumers’ point of view [15].

In view of the above, it was decided to verify whether a confectionary product such as muffins may be enriched with bioactive compounds, e.g., omega-3 and omega-6 acids, plant sterols, phenolic compounds, and carotenoids. An excellent source of all three of these groups of compounds may be provided by camelina oil [16,17], an oil traditionally cold-pressed from the seeds of camelina (*Camelina sativa*). This plant is an annual oil crop belonging to the family *Brassicaceae* and has been used for 3000 years to produce oil [12]. Camelina oil was entered into the register of traditional products in the Wielkopolskie province (Poland) in 2006. Camelina oil is found in a wide variety of colours, ranging from golden (straw-coloured) to reddish-brown. The oil colour depends on the variety of camelina used (spring vs. winter) and the applied seed heating method [18]. Camelina oil is characterised by an onion or mustard aftertaste, as well as a strong, distinct aroma. It has a longer shelf life in contrast to other oils with a comparable composition and ratios of saturated and unsaturated fatty acids (SFAs/UFAs) [19], connected to its high contents of natural antioxidants, such as tocopherols, phenolic acids, and flavonoids [20]. To date, analyses of camelina oil have focused primarily on tocopherols [21]. Earlier studies [6,12,22] showed that in contrast to other oils, camelina oil is also characterised by high contents of phenolic acids and flavonoids, as well as carotenoids. The fatty acid composition in this oil is advantageous from a nutritional point of view [7,23]. The omega-3 to omega-6 ratio (2:1) means that it may be considered a special purpose functional food, i.e., a dietary supplement enriching the diet mainly with omega-3 acids [24,25,26,27,28].

Camelina seeds have identical properties to those reported for camelina oil, but additionally contain large amounts of soluble dietary fibre fraction. Dietary fibre aids the treatment of constipation, as it stimulates the differentiation and proliferation of intestinal epithelial cells, promotes proliferation of beneficial intestinal microbiota and maintenance of an optimal pH in the gut, and also plays a role in the prevention of colon cancer [29]. However, camelina seeds also contain antinutritional substances, such as small amounts of phytic acid and high levels of tannins [30]. Although tannins are considered antinutritional compounds, they may exhibit health-promoting properties [31]. They limit the growth of cancer cells, primarily thanks to their antioxidative activity. Moreover, tannins exhibit antibacterial activity and are capable of modifying the microflora of the oral cavity and the gut by eliminating pathogenic organisms. From the consumers’ point of view, it is important that tannins provide products with a bitter taste, which may be considered desirable. Phytic acid is one of the most fascinating bioactive food compounds and is widely distributed in plant foods. This acid exhibits a variety of properties and may exert diverse effects on humans and animals. Due to its molecular structure, phytic acid shows high affinity to minerals and disturbs intestinal absorption. However, in a well-balanced diet, this problem seems to have less of an impact. In developed countries with a high incidence of various lifestyle diseases, the advantageous properties of phytic acids, such as its antioxidative and anti-carcinogenic properties, are of considerable importance [32].

The aforementioned properties of both camelina oil and camelina seeds predispose them to applications in the development of various food products.

The aim of this study was to see whether it is possible to add camelina oil and camelina seeds as ingredients in model muffins in order to enhance their health-promoting value, such as the content of bioactive compounds, while maintaining the organoleptic attributes considered desirable by consumers.

## 2. Material and Methods 

### 2.1. Experimental Material 

Analyses were conducted on oil pressed from seeds of a spring camelina cultivar, Omega. This cultivar was developed by the Department of Genetics and Plant Breeding (KGiHR) at the Poznań University of Life Sciences and since 2013, has been protected by National Plant Breeders’ Rights in Poland (National PBR). Camelina seeds were harvested from fields of the agricultural research station in Dłoń, belonging to the Poznań University of Life Sciences (51°41′23.835″ N, 17°4′1.414″ E). Oil was pressed using a machine line for efficient oil pressing from camelina seeds, manufactured as part of a research project under the EUREKA International Scientific Program “E!4018 CAMELINA-BIOFUEL” at the Industrial Institute of Agricultural Engineering in Poznań. This set of machines comprises a screw press, a crusher, and a screw conveyor facilitating the continuous cold-pressing of oil from oil crop seeds. Cold pressing of oil takes place at a temperature no higher than 40–50 degrees Celsius. The oil production efficiency is 89% and the press capacity is 100 kg/hour [6].

Commercially available virgin rapeseed oil, 100% refined and cold filtered, was purchased from a retail chain store.

The control (M*Bn*O) consisted of muffins prepared from components typically used for muffin preparation, i.e., 27.69% wheat flour, 25.47% milk (2% fat), 22.14% sugar, 12.4% eggs, 11.85% rapeseed oil, and 0.45% baking powder. In muffins with an addition of camelina oil (M*Cs*O), the entire volume of rapeseed oil was substituted with oil pressed from camelina seeds. In order to prepare the muffin variants with an addition of camelina seeds, the dough for the M*Bn*O and M*Cs*O variants was supplemented with 6.65% camelina seeds in relation to the mass of prepared dough. The muffins were labelled M*Cs*S (the variant with an addition of camelina seeds) and M*Cs*OS (muffins with an addition of both camelina oil and camelina seeds).

The dough (60 g) was placed into silicon muffin cups and baked in a preheated oven at 180 °C for 25 min. After baking, the muffins were cooled to room temperature and packed in polypropylene bags. Sensory analysis was performed on the same day, while the muffins for chemical analyses were frozen.

All chemical analyses described below (FAME analysis, determination of phytosterols, carotenoids, and phenolic compounds) were performed for the camelina seeds (*Cs*S), camelina oil (*Cs*O), rapeseed oil (*Rs*O), and model muffins (M*Bn*O, M*Cs*O, M*Cs*S, M*Cs*OS).

### 2.2. FAME Analysis

The fatty acid profile was determined in the analysed cultivars to characterise the lipid fraction as a potential source of flavour/volatile compounds. Fatty acids were extracted using a method described by Stuper-Szablewska, Buśko, Góral, and Perkowski [33]. Samples containing 100 mg of ground grains were placed into 17 mL culture tubes, suspended in 2 mL of methanol, treated with 0.5 mL of 2 M aqueous sodium hydroxide, and tightly sealed. The culture tubes were then placed within 250 mL plastic bottles, sealed tightly, and placed inside a microwave oven (Model AVM 401/1WH; Whirlpool, Stockholm Sweden) operating at 2450 MHz and 900 W maximum output. Samples were irradiated (370 W) for 20 s and after approx. 5 min, for an additional 20 s. After 15 min, the contents of the culture tubes were neutralised with 1 M aqueous hydrochloric acid; next, 2 mL MeOH were added and extraction with pentane (3–4 mL) was carried out within the culture tubes. The combined pentane extracts were evaporated to dryness in a nitrogen stream. In the next step, extracts were methylated using a mixture of anhydrous methanol and sulfuric acid (1:5, *v*/*v*). The extract containing the lipids was supplemented with 0.5 mL of methanol followed by the addition of a 0.15 mL methanol/sulfuric acid mixture (1:5, *v*/*v*). The samples were heated at 70 °C for 15 min. After the solution had been cooled, 0.5 mL of n-hexane was added, followed by the addition of sufficient water to form two layers. The upper hexane layer was removed and analysed on an Aquity H class UPLC system equipped with a Waters Acquity PDA detector (Waters, Milford, MA, USA). Chromatographic separation was performed on an Acquity UPLC^®^ BEH C_18_ column (150 mm × 2.1 mm, particle size 1.7 μm) (Waters, Dublin, Ireland). The elution was carried out in the gradient using the following mobile phase composition: A, acetonitrile; B, 2-propanol, and a flow rate of 0.17 mL/min. Measurements of sterol concentrations were performed using an external standard at wavelengths λ = 195–300. Compounds were identified based on a comparison of retention times for the examined peak with that of the standard, and by adding a specific amount of the standard to the tested sample and repeating the analyses. The limit of detection was 0.01 mg/kg.

### 2.3. Determination of Phytosterols

Sterols were determined following microwave-assisted basic hydrolysis. Samples of 100 mg ground material were placed into 17 mL culture tubes, suspended in 1 mL of methanol, treated with 0.1 mL of 2 M aqueous NaOH, and sealed tightly. Next the culture tubes were placed within 250 mL plastic bottles, sealed tightly, and placed inside a microwave oven (Whirlpool model AVM 401/WH) operating at 2450 MHz and 900 W maximum output. Samples were irradiated (370 W) for 20 s, then, after c. 5 min, for an additional 20 s and extracted with pentane (HPLC grade, Sigma-Aldrich, Steinheim, Germany) (3 × 4 mL) within the culture tubes. The combined pentane extracts were evaporated to dryness in a gentle stream of a high purity nitrogen using a RapidVap Evaporator (Labconco, Kansas, MO, USA). The extracts were stored at −25 °C until analysis. Prior to analysis, samples were dissolved in 1 mL of methanol, filtered through 13 mm syringe filters with a 0.22 μm pore diameter (Fluoropore Membrane Filters, Sigma Aldrich Sp. z o.o., Poznań, Poland). The contents of sterols were analysed using an Aquity H class UPLC system equipped with a Waters Acquity PDA detector (Waters, Milford, MA, USA). Chromatographic separation was performed on an Acquity UPLC^®^ BEH C_18_ column (100 mm × 2.1 mm, particle size 1.7 μm) (Waters, Dublin, Ireland). The elution was carried out isocratically using the following mobile phase composition: A, acetonitrile 10%; B, methanol 85%; C, water 5%, at a flow rate of 0.5 mL/min. Sterol concentrations were determined using an external standard at the wavelength λ = 210 (campesterol, delta 5-avenasterol, brassicasterol, stigmasterol, β-sitosterol). The compounds were identified based on a comparison of the retention times of the examined peak with those of the standard, and by adding a specific amount of the standard to the tested sample and repeating the analyses. The limit of detection was 0.1 mg/kg [34].

### 2.4. Determination of Carotenoids

Carotenoid isolation and quantification in grain samples were performed by the saponification method using an Acquity UPLC apparatus (Waters, Milford, MA, USA). Carotenoid extracts were obtained from ground seeds (0.4 mg), which were triturated with a mixture of acetone and petroleum ether (1:1). Following separation of the plant tissue, the acetone and the hydrophilic fraction were removed from the extract by washing with water. As a result, the ether extract was obtained with a mixture of carotenoid pigments. The prepared extract was concentrated in a vacuum evaporator at 35 °C until an oily residue was obtained, then digested in 2 mL of methanol (Sigma Aldrich Sp. z o.o., Poznań, Poland) and subjected to chromatographic analysis. Lutein, zeaxanthin, and β-carotene were determined using an Acquity UPLC system (Waters, Milford, MA, USA) with a Waters Acquity PDA detector (Waters, Milford, MA, USA). Chromatographic separation was performed on an Acquity UPLC^®^ BEH C18 column (100 mm × 2.1 mm, particle size 1.7 μm) (Waters, Dublin, Ireland). Elution was carried out using solvent A, methanol; B, water, and tert-butyl methyl ether (TBME). A gradient was applied at a flow rate of 0.4 mL/min. The column and samples were measured with a thermostat, the column temperature was 30 °C and the test temperature was 10 °C. During the analysis, the solutions were degassed in a Waters device. The injection volume was 10 μL. The values were recorded at a wavelength of λ = 445 nm. Respective compounds, i.e., lutein, zeaxanthin, and beta-carotene, were identified based on spectra ranging from 200 to 600 nm and retention times compared to the standards [22].

### 2.5. Determination of Phenolic Compounds

Determination of phenolic compounds was conducted in accordance with the methodology described by Stuper-Szablewska, Kurasiak-Popowska, Nawracała and Perkowski. The detection level was 1 μg/g. The retention times of the assayed acids are as follows: kaempferol, 6.11 min; gallic acid, 8.85 min; vanilic acid, 9.71 min; luteolin, 11.89 min; protocatechuic acid, 12.23 min; vanillin, 14.19 min; apigenin, 16.43 min; catechin, 18.09 min; 4-hydroxybenzoic acid, 19.46 min; chlorogenic acid, 21.56 min; caffeic acid, 26.19 min; syringic acid, 28.05 min; naringenin, 31.22 min; vitexin, 35.41 min; rutin, 38.11 min; quercetin, 39.58 min; p-coumaric acid, 40.20 min; ferulic acid, 46.20 min; synapic acid, 48.00 min, and t-cinnamic acid, 52.40 min [35].

### 2.6. Sensory Analysis

**Figure 1:**
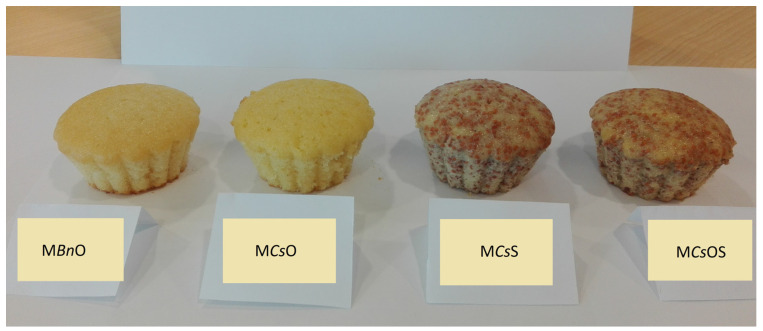
Model muffins with the addition of *Camelina sativa* seed and oil. Sample codes: M*Bn*O—control muffins (containing rapeseed oil), M*Cs*O—muffins containing camelina oil, M*Cs*S—muffins containing camelina seeds, M*Cs*OS—muffins containing camelina oil and camelina seeds.

The sensory analysis was performed by a panel of specially selected individuals. All panellists had been trained in sensory analysis. Their evaluation ability was checked using a control card. Conditions for the performance of sensory evaluation met the requirements of the standards concerning the methodology and procedures of quality analyses for bakery and confectionary products. The material for sensory analyses comprised baked muffins cooled to room temperature. The following muffins were used for the analysis: M*Bn*O—muffins produced with rapeseed oils, M*Cs*O—muffins produced with camelina oil, M*Cs*S—muffins containing camelina seeds, and M*Cs*OS—muffins containing camelina oil and camelina seeds. All model muffins are shown on Figure 1. Sensory analyses included quality attributes such as colour, taste, aroma, texture, and appearance. Overall desirability was also evaluated. 

The ratings were made on a 10-point hedonic scale, ranging from 10 (like extremely) to 1 (dislike extremely) for each attribute [36].

### 2.7. Statistical Analysis

All analytical values represent the means of three analyses performed in at least two different experiments. 

To ensure the objectivity of the obtained results, the data were subjected to statistical verification. Statistical analysis was conducted using the Statistica 13.3 programme. In the first stage of statistical analysis, means, standard deviations, confidence intervals, and statistical errors were calculated. To verify the normality of distributions the Kolmogorov–Smirnov test with the Lilliefors correction and the Shapiro–Wilk W-test were performed. The next step in statistical analysis included one-way analysis of variance (ANOVA) (*p* < 0.05) using Cochran’s C-test, Hartley’s test, and Barlett’s test. Next, for parametric data, Tukey’s post-hoc Honest Significance Difference (HSD) test was performed.

## 3. Results and Discussion

Within this study, the analyses were conducted on model muffins produced with the addition of camelina oil (M*Cs*O) or camelina seeds (M*Cs*S), as well as both camelina oil and camelina seeds (M*Cs*OS). Recorded results were compared with those of the control, i.e., muffins produced with rapeseed oil (M*Bn*O). Additionally, the camelina oil (*Cs*O), rapeseed oil (*Bn*O), and camelina seeds (*Cs*S) used in the production of the model muffins were also analysed in terms of their bioactive compound contents. 

In all the above-mentioned samples, the fatty acid profile was determined (Table 1). 

Camelina oil was characterised by a high content of linolenic acid (C18:3ω-3), which was four-fold greater than in rapeseed oil. A similar composition of fatty acids was assayed in the seeds. The fatty acid profile in the baked model muffins changed significantly in comparison to that in the raw material. In the muffins containing rapeseed oil, a high increase was recorded for the shares of saturated fatty acids such as C16:0, C18:0, C20:0, and C15:0, as well as monounsaturated C18:1. The change in the fatty acid profile of the muffins with the addition of camelina oil was significant; however, it was found that as a result of thermal treatment, lower amounts of saturated fatty acids were formed. An exception in this respect was reported for C17:0. The analyses showed that muffins with the addition of camelina oil contained higher amounts of unsaturated fatty acids compared to the control. In muffins with the addition of seeds, the fatty acid profile was comparable to that of muffins with camelina oil. The content of C20:1 was approx. six-fold greater in muffins with camelina oil. In turn, the level of C20:0 was very low compared with the control muffins or muffins with camelina oil. An almost two-fold increase was recorded in the content of C18:2ω-6. Additionally, C16:1 and C18:0 were found in slight amounts, approx. 1%, when compared to the control or the muffins with the camelina oil added. The last analysed variant of muffins, i.e., those with camelina seeds and camelina oil instead of rapeseed oil, did not differ significantly from the muffins with camelina oil in terms of their fatty acid profile, while a higher C20:0 content was recorded compared to the muffins with camelina seeds and those with camelina oil. Phytosterols comprised another group of bioactive compounds exhibiting a health-promoting effect and were thus analysed in this study. Analyses were conducted on the five most important sterols found in oil crops (Table 2). The rapeseed oil and the muffins with the rapeseed oil added were characterised by higher contents of brassicasterol. Both camelina oil, camelina seeds, and muffins with both of these addition contained two- to seven-fold lower amounts of this sterol. Thermal treatment resulted in an average 6% decrease in phytosterols, in comparison to the input materials. To date, no studies have been conducted on changes in the sterol contents in camelina oil during heating; nevertheless, the results may be compared with those concerning rapeseed oil. For example, the application of 180 °C for 10 min during frying leads to approx. 5% sitosterol losses in rapeseed oil with sitosterol esters added [37]. The decrease in phytosterol contents during the thermal treatment of oils is dependent on many factors, such as the process conditions or the presence of antioxidants and oxidative substances [38]. Moreover, it was shown that considerable losses of phytosterols are also observed during the pressing and refining of oils [39]. Analyses conducted within this study showed that camelina oil and camelina seeds are rich in delta 5-avenasterol, the level of which was two-fold higher than in rapeseed oil. The concentrations of β-sitosterol were similar in all tested samples, with the highest amounts of this compound recorded in muffins with the addition of camelina oil and camelina seeds.

Carotenoid contents are dependent on the ripeness of the oil crop seeds used to produce oil. The quality of oil was determined based on its colour, specified in two ways; either analysis of the total carotenoid pigments, or analysis of the chlorophyll content. To date, literature on the subject has focused on the analyses of tocochromanols [40,41,42,43,44,45]. The performed pilot studies showed that camelina oil is also rich in carotenoid pigments [22]. Next to beta-carotene, the level of which is over 50% higher in camelina oil than in rapeseed oil, the contents of zeaxanthin and lutein were three-fold greater compared to rapeseed oil. In comparison to the raw material, the application of thermal treatment considerably reduced the carotenoid contents in the product by 50% on average, depending on the characteristics of the compound (Table 3).

The last analysed group of bioactive compounds comprised phenolic compounds. The contents of eight flavonoid aglycones and 12 phenolic acids were analysed (Table 4). There were statistically significant differences between the contents of these compounds in rapeseed oil compared to camelina oil or camelina seeds. Differences observed in the raw material were also reflected in the model muffins. Among all the investigated experimental variants, muffins produced with the addition of camelina seeds and camelina oil were characterised by the highest contents of all the analysed phenolic acids. Thermal processing caused a significant decrease in phenolic acids when compared to the raw material; while in the case of flavonoids, temperature caused no significant decrease in the contents of these compounds.

It is known that high levels of added compounds in food products may be significantly involved in the sensations of taste and aroma. Moreover, by adding some solid particles such as seeds into dough, the texture can be changed. The change in oil can also affect the texture of the product [46].

**Figure 2:**
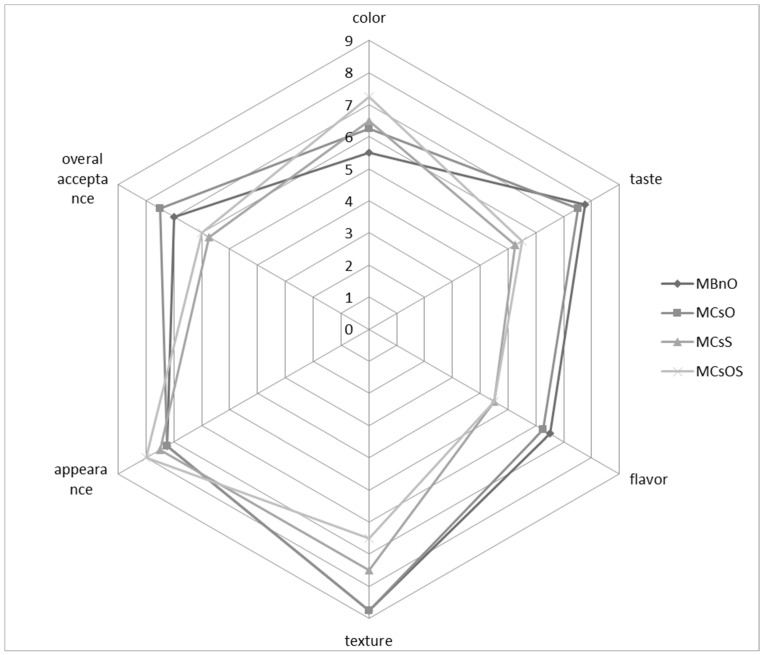
Radar plots of sensory data for muffins with the addition of *Camelina sativa* seeds and oil. Sample codes: M*Bn*O—control muffins (containing rapeseed oil), M*Cs*O—muffins containing camelina oil, M*Cs*S—muffins containing camelina seeds, M*Cs*OS—muffins containing camelina oil and camelina seeds.

Sensory analyses of muffins were conducted in order to verify whether and to what extent an addition of camelina oil or camelina seeds influenced quality attributes. Figure 2 presents radar plots for the sensory data of muffins produced with all of the typically used ingredients (M*Bn*O), as well as muffins with the addition of camelina oil (M*Cs*O), camelina seeds (M*Cs*S), and both camelina oil and camelina seeds (M*Cs*OS). The sensory evaluation of the muffin samples showed that the addition of *Camelina sativa* seeds increased the scores for colour and appearance, but reduced the scores for taste, flavour, texture, and overall acceptance when compared to the control muffins. The addition of seeds and both seeds and oil slightly worsened the evaluation of texture. No statistically significant differences were recorded between the control muffins (M*Bn*O) and the muffins with the addition of camelina oil (M*Cs*O). Replacing rapeseed oil with camelina oil did not change the texture. The muffins with camelina oil received high scores comparable to those of the control muffins, and it was stated that muffins with camelina oil had a more advantageous colour, which contributed to their greater overall desirability.

## 4. Conclusions

The substitution of rapeseed oil with camelina oil had no negative effect on most of the organoleptic attributes of muffins. Moreover, thanks to the greater contents of carotenoids, camelina oil had an advantageous effect on the product colour, thus improving its overall desirability. An attempt to increase the share of bioactive compounds in the muffins by supplementing them with camelina seeds resulted in a deterioration of the scores for most evaluated quality attributes, although the scores (all exceeding the mean) still indicated that the product is sensorily acceptable.

In conclusion, *Camelina sativa* seeds and oil may be successfully used as ingredients in muffins in order to enhance their health-promoting value.

## Figures and Tables

**Table 1:** Fatty acid contents in the raw material and in muffins (% *w*/*w*).

Fatty Acids		*Bn*O	*Cs*O	*Cs*S	M*Bn*O	M*Cs*O	M*Cs*S	M*Cs*OS
Formula	Name (Acronym)							
**C14:0**	Myristic acid	0.00	0.20	0.10	0.00	0.00	0.00	0.00
**C15:0**	Pentadecanoic acid	0.00	0.00	0.00	0.60	0.00	0.00	0.00
**C15:1**	Pentadecenoic acid	0.00	0.20	0.10	0.00	0.00	0.00	0.00
**C16:0**	Palmitic acid (PA)	4.40	5.90	5.10	19.23	13.26	8.23	5.39
**C16:1**	Palmitoleic acid	0.20	0.80	0.60	0.00	0.00	0.10	0.00
**C17:0**	Margaric acid	0.10	0.10	0.10	18.23	30.25	23.15	26.23
**C17:1**	10-heptadecenoic acid	0.00	0.10	0.20	0.00	0.00	0.00	0.00
**C18:0**	Stearic acid	1.70	2.10	2.10	8.55	0.00	1.06	0.65
**C18:1**	Oleic acid	64.23	13.52	13.2	35.63	14.06	10.22	3.56
**C18:2ω-6**	Linoleic acid (LA)	16.20	17.90	16.3	4.58	5.13	12.52	4.36
**C18:3ω-6**	y-Linolenic acid (GLA)	0.10	0.30	0.10	0.00	0.00	0.00	0.00
**C18:3ω-3**	α- Linolenic acid (ALA)	9.20	36.02	35.9	1.36	26.22	26.36	29.66
**C20:0**	Arachidic acid (ARA)	0.40	4.60	1.30	12.36	8.25	0.60	10.23
**C20:1**	c-11-eicosenoic acid	1.20	15.63	24.4	0.00	3.25	18.56	20.56
**C20:2**	c-11, 14-eicosadienoic acid	0.10	0.10	0.50	0.00	0.00	0.00	0.00
**C22:1**	Erucic acid (EU)	0.30	2.60	0.90	0.00	0.30	0.00	0.00
**C24:0**	Lignoceric acid	0.30	0.10	0.00	0.00	0.00	0.00	0.00
**C24:1**	Nervonic acid	0.00	0.70	0.00	0.00	0.00	0.00	0.00

Values bearing different superscripts are statistically significantly different (*p* ≤ 0.05). *Bn*O—rapeseed oil, *Cs*O—camelina oil, *Cs*S—camelina seeds, M*Bn*O—muffins produced with rapeseed oils, M*Cs*O—muffins produced with camelina oil, M*Cs*S—muffins containing camelina seeds, M*Cs*OS—muffins containing camelina oil and camelina seeds.

**Table 2:** Phytosterol contents in the raw material and in the muffins (mg/kg).

	*Bn*O	*Cs*O	*Cs*S	M*Bn*O	M*Cs*O	M*Cs*S	M*Cs*OS
**Campesterol**	271.3 ± 1.58 ^b^	120.3 ± 0.08 ^a^	118.2 ± 0.91 ^a^	259.4 ± 1.29 ^c^	116.5 ± 1.01 ^b^	106.4 ± 0.88 ^a^	146.4 ± 1.02 ^b^
**Stigmasterol**	3.5 ± 0.02 ^b^	1.1 ± 0.01 ^a^	ND	2.0 ± 0.02 ^b^	0.6 ± 0.01 ^a^	ND	1.9 ± 0.02 ^b^
**β-sitosterol**	356.4 ± 1.98 ^ab^	385.2 ± 2.11 ^b^	321.6 ± 2.04 ^a^	321.3 ± 2.13 ^a^	369.0 ± 2.19 ^b^	305.2 ± 1.86 ^a^	456.4 ± 2.56 ^c^
**delta 5-avenasterol**	26.6 ± 0.03 ^a^	46.4 ± 0.04 ^ab^	57.3 ± 0.04 ^b^	26.5 ± 0.04 ^a^	37.6 ± 0.04 ^b^	54.2 ± 0.05 ^c^	66.5 ± 0.06 ^d^
**Brassicasterol**	70.3 ± 0.04 ^c^	10.3 ± 0.03 ^a^	27.0 ± 0.03 ^b^	59.4 ± 0.06 ^c^	8.7 ± 0.02 ^a^	20.4 ± 0.04 ^b^	25.3 ± 0.03 ^b^

Values (mean ± SD of three replicates) bearing different superscripts are statistically significantly different (*p* ≤ 0.05). ND—not detected, *Bn*O—rapeseed oil, *Cs*O—camelina oil, *Cs*S—camelina seeds, M*Bn*O—muffins produced with rapeseed oils, M*Cs*O—muffins produced with camelina oil, M*Cs*S—muffins containing camelina seeds, M*Cs*OS—muffins containing camelina oil and camelina seeds.

**Table 3:** Carotenoid contents in the raw material and in the muffins (mg/kg).

	*Bn*O	*Cs*O	*Cs*S	M*Bn*O	M*Cs*O	M*Cs*S	M*Cs*OS
**Lutein**	5.12 ± 0.02 ^a^	14.58 ± 0.08 ^b^	16.10 ± 0.03 ^b^	2.16 ± 0.02 ^a^	6.23 ± 0.02 ^b^	8.22 ± 0.01 ^b^	13.52 ± 0.09 ^c^
**Zeaxanthin**	2.05 ± 0.02 ^a^	6.54 ± 0.03 ^b^	7.09 ± 0.04 ^b^	0.66 ± 0.01 ^a^	2.33 ± 0.02 ^b^	5.62 ± 0.03 ^b^	9.25 ± 0.09 ^c^
**Beta-carotene**	89.36 ± 0.41 ^a^	120.74 ± 0.59 ^b^	133.69 ± 0.60 ^b^	43.25 ± 0.26 ^a^	79.58 ± 0.39 ^b^	111.36 ± 0.42 ^c^	149.65 ± 0.62 ^d^

Values (mean ± SD of three replicates) bearing different superscripts are statistically significantly different (*p* ≤ 0.05). *Bn*O—rapeseed oil, *Cs*O—camelina oil, *Cs*S—camelina seeds, M*Bn*O—muffins produced with rapeseed oils, M*Cs*O—muffins produced with camelina oil, M*Cs*S—muffins containing camelina seeds, M*Cs*OS—muffins containing camelina oil and camelina seeds.

**Table 4:** Contents of phenolic compounds in the raw material and in the muffins (mg/kg).

		*Bn*O	*Cs*O	*Cs*S	M*Bn*O	M*Cs*O	M*Cs*S	M*Cs*OS
**Flavonoid aglycones**	Apigenin	26.36 ± 0.51 ^a^	85.24 ± 0.91 ^b^	94.62 ± 1.07 ^b^	20.16 ± 0.52 ^a^	80.74 ± 0.61 ^b^	89.65 ± 0.61 ^b^	116.85 ± 1.02 ^c^
	Catechin	2.45 ± 0.05 ^a^	7.26 ± 0.06 ^b^	9.23 ± 0.10 ^b^	1.82 ± 0.04 ^a^	6.89 ± 0.08 ^b^	8.16 ± 0.11 ^b^	12.52 ± 0.16 ^b^
	Kaempferol	16.22 ± 0.24 ^a^	48.25 ± 0.71 ^b^	40.45 ± 0.65 ^b^	14.52 ± 0.21 ^a^	39.56 ± 0.71 ^b^	36.75 ± 0.65 ^b^	61.25 ± 0.92 ^c^
	Luteolin	10.25 ± 0.15 ^a^	55.25 ± 0.76 ^b^	107.09 ± 0.98 ^c^	8.98 ± 0.10 ^a^	49.52 ± 0.73 ^b^	89.99 ± 1.33 ^c^	146.52 ± 2.01 ^d^
	Naringenin	35.23 ± 0.56 ^a^	59.25 ± 0.82 ^b^	86.39 ± 1.01 ^c^	26.56 ± 0.39 ^a^	47.12 ± 0.71 ^b^	72.35 ± 1.01 ^c^	119.85 ± 2.35 ^d^
	Quercetin	29.56 ± 0.32 ^a^	119.25 ± 1.03 ^c^	46.69 ± 0.42 ^b^	25.85 ± 0.35 ^a^	100.52 ± 1.42 ^b^	32.52 ± 0.48 ^a^	126.56 ± 1.76 ^c^
	Rutin	6.58 ± 0.07 ^a^	10.41 ± 0.02 ^a^	34.01 ± 0.03 ^b^	5.22 ± 0.07 ^a^	8.96 ± 0.12 ^a^	26.85 ± 0.41 ^b^	33.56 ± 0.36 ^c^
	Vitexin	29.36 ± 0.51 ^a^	50.41 ± 0.91 ^b^	41.96 ± 0.82 ^b^	24.13 ± 0.48 ^a^	42.16 ± 0.79 ^b^	35.69 ± 0.69 ^ab^	70.45 ± 1.26 ^c^
**Phenolic acids**	4-hydroxybenzoic	31.25 ± 0.46 ^a^	52.14 ± 0.52 ^b^	174.12 ± 1.68 ^c^	6.56 ± 0.11 ^a^	14.52 ± 0.24 ^b^	36.52 ± 0.61 ^c^	39.52 ± 0.66 ^c^
	Caffeic	28.63 ± 0.31 ^a^	106.24 ± 1.21 ^b^	149.93 ± 1.92 ^c^	5.84 ± 0.12 ^a^	12.63 ± 0.24 ^b^	26.56 ± 0.50 ^c^	45.69 ± 0.85 ^d^
	Chlorogenic	56.36 ± 0.57 ^a^	165.33 ± 1.62 ^c^	149.61 ± 1.35 ^b^	16.36 ± 0.26 ^a^	46.52 ± 0.91 ^c^	36.22 ± 0.72 ^b^	59.85 ± 1.26 ^d^
	Ferulic	28.55 ± 0.50 ^a^	46.37 ± 0.72 ^b^	92.42 ± 1.65 ^c^	6.85 ± 0.07 ^a^	10.52 ± 0.12 ^a^	24.25 ± 0.27 ^b^	30.56 ± 0.29 ^c^
	Gallic	10.45 ± 0.15 ^a^	12.33 ± 0.15 ^a^	25.97 ± 0.21 ^b^	2.41 ± 0.03 ^a^	3.45 ± 0.03 ^a^	6.52 ± 0.06 ^b^	8.98 ± 0.11 ^c^
	p-Coumaric	1.06 ± 0.01 ^a^	4.12 ± 0.02 ^b^	13.62 ± 0.09 ^c^	0.16 ± 0.01 ^a^	1.65 ± 0.02 ^a^	4.85 ± 0.06 ^b^	6.89 ± 0.06 ^c^
	Protocatechuic	44.52 ± 0.81 ^a^	65.33 ± 1.17 ^b^	181.02 ± 3.26 ^c^	16.36 ± 0.31 ^a^	21.63 ± 0.39 ^b^	68.12 ± 0.75 ^c^	72.66 ± 0.82 ^c^
	Sinapic	76.36 ± 0.77 ^a^	65.302 ± 1.25 ^b^	107.86 ± 3.25 ^c^	21.36 ± 0.39 ^a^	65.25 ± 0.65 ^b^	119.63 ± 1.21 ^c^	100.85 ± 1.00 ^c^
	Syringic	33.52 ± 0.45 ^a^	63.24 ± 0.72 ^b^	115.71 ± 1.11 ^c^	13.25 ± 0.21 ^a^	15.62 ± 0.21 ^a^	36.54 ± 0.35 ^b^	49.85 ± 0.52 ^c^
	t-Cinnamic	37.42 ± 0.40 ^a^	52.33 ± 0.49 ^b^	104.99 ± 0.98 ^c^	14.79 ± 0.20 ^a^	22.41 ± 0.25 ^b^	38.52 ± 0.41 ^c^	60.45 ± 0.72 ^d^
	Vanillin	26.56 ± 0.36 ^a^	59.37 ± 0.62 ^b^	30.90 ± 0.39 ^a^	4.69 ± 0.06 ^a^	12.36 ± 0.15 ^b^	6.36 ± 0.09 ^ab^	26.52 ± 0.51 ^c^
	Vanillic acid	3.45 ± 0.03 ^b^	0.70 ± 0.01 ^a^	1.85 ± 0.02 ^a^	10.55 ± 0.15 ^b^	6.85 ± 0.07 ^a^	16.85 ± 0.20 ^c^	34.16 ± 0.49 ^d^

Values (mean ± SD of three replicates) bearing different superscripts are statistically significantly different (*p* ≤ 0.05). *Bn*O—rapeseed oil, *Cs*O—camelina oil, *Cs*S—camelina seeds, M*Bn*O—muffins produced with rapeseed oils, M*Cs*O—muffins produced with camelina oil, M*Cs*S—muffins containing camelina seeds, M*Cs*OS—muffins containing camelina oil and camelina seeds.

## Data Availability

The original contributions presented in the study are included in the article, further inquiries can be directed to the corresponding author.
